# Mealworm-Derived Protein Hydrolysates Enhance Adipogenic Differentiation via Mitotic Clonal Expansion in 3T3-L1 Cells

**DOI:** 10.3390/foods14020217

**Published:** 2025-01-12

**Authors:** Hee-Jeong Ryu, Syng-Ook Lee

**Affiliations:** Department of Food Science and Technology, Keimyung University, Daegu 42601, Republic of Korea; uhee9655@naver.com

**Keywords:** adipogenesis, adiponectin, flavourzyme, mealworms, *Tenebrio molitor*, 3T3-L1

## Abstract

Adipocytes secrete adipokines, bioactive molecules crucial for various physiological processes, such as enhancing insulin sensitivity, promoting wound healing, supporting hair growth, and exhibiting anti-aging effects on the skin. With the growing global demand for sustainable and alternative protein sources, insect-derived proteins, particularly from *Tenebrio molitor* (mealworms), have gained attention due to their high nutritional value and functional bioactivities. This study aims to explore the potential of mealworm-derived protein hydrolysates as novel bioactive materials for promoting adipogenesis and improving adipokine expression, with applications in metabolic health and skin regeneration. Protein hydrolysates (<1 kDa) were prepared using enzymatic hydrolysis with three proteases (alcalase, flavourzyme, and neutrase) and evaluated for their adipogenic activity in 3T3-L1 preadipocytes. Among them, the flavourzyme-derived hydrolysate (Fh-T) exhibited the most significant effects, enhancing adipogenic differentiation and lipid accumulation. Fh-T facilitated adipogenesis by promoting mitotic clonal expansion (MCE) during the early stage of differentiation, which was associated with the upregulation of C/EBPδ and the downregulation of p27. These findings underscore the potential of mealworm-derived protein hydrolysates, particularly Fh-T, as sustainable and functional ingredients for use in glycemic control, skin health, and tissue regeneration. This study provides valuable insights into the innovative use of alternative protein sources in functional foods and cosmeceuticals.

## 1. Introduction

Adipose tissue, once regarded merely as an energy storage site, is now recognized for its multifaceted functions that significantly impact human health [1,2]. Adipocytes, the primary cells of adipose tissue, secrete adipokines, bioactive molecules that regulate key physiological processes. Among them, adiponectin plays a critical role in enhancing insulin sensitivity by promoting glucose uptake and fatty acid oxidation while suppressing hepatic gluconeogenesis. Reduced adiponectin levels are strongly associated with insulin resistance, obesity, and type 2 diabetes [3], highlighting its importance in metabolic health.

Subcutaneous fat, the primary white adipose tissue located beneath the dermis, performs additional roles beyond energy storage. It helps regulate body temperature, maintains skin elasticity, and mitigates wrinkle formation. Moreover, adipocytes contribute to wound healing by secreting critical cytokines and growth factors, including fibroblast growth factors, insulin-like growth factors, epidermal growth factors, and vascular endothelial growth factors [2]. Adipokines like adiponectin and *Leptin* also maintain skin integrity and stimulate collagen synthesis in dermal fibroblasts. Furthermore, these adipokines promote hair growth in human follicles, demonstrating their regenerative potential [4,5].

Meanwhile, with the growing demand for sustainable and high-quality protein sources, edible insects have gained significant attention. Among them, *Tenebrio molitor* larvae (mealworm) are particularly promising due to their high protein content, essential fats, and micronutrients [6,7,8]. In addition to their nutritional value, mealworms present a sustainable solution to the increasing challenges of food security and environmental impact associated with conventional livestock production.

Protein hydrolysates derived from mealworms have been shown to exhibit diverse bioactivities, including antioxidant, hair growth-promoting, and ACE inhibitory effects [9,10,11]. These bioactivities are largely attributed to biologically active peptides released during enzymatic hydrolysis. The process of enzymatic hydrolysis using proteases involves breaking down proteins into smaller peptide fragments and amino acids, which can enhance the functional properties of the resulting hydrolysates. Active peptides isolated through this process have been reported to improve physiological functions by targeting specific pathways, including those involved in metabolic health and cellular differentiation.

Building on prior research demonstrating the antioxidant properties of mealworm protein hydrolysates [6,12], this study investigates their ability to stimulate adipogenic differentiation and enhance adiponectin expression in 3T3-L1 preadipocytes. By utilizing protein hydrolysates derived from mealworms, an innovative and sustainable resource, this research underscores their novelty in promoting adipogenesis. While the bioactivities of insect-derived materials have gained increasing attention, the potential of mealworm protein hydrolysates, particularly in enhancing adipogenic differentiation and adipokine expression, remains largely unexplored. Through an investigation of their mechanisms and effects, this study provides new insights into the application of mealworm-derived ingredients as functional materials in metabolic health and skin regeneration.

## 2. Materials and Methods

### 2.1. Materials

The 3T3-L1 preadipocyte cell line was obtained from the American Type Culture Collection (Rockville, MD, USA). Cells were maintained in high-glucose Dulbecco’s Modified Eagle Medium (DMEM) (Welgene, Gyeongbuk, Republic of Korea) supplemented with 10% bovine calf serum, 100 units/mL penicillin, and 100 µg/mL streptomycin in a humidified atmosphere of 5% CO_2_ at 37 °C. Primary antibodies for C/EBPα, C/EBPβ, C/EBPδ, PPARγ, adiponectin, GAPDH, and anti-rabbit polyclonal antibodies were sourced from Cell Signaling Technology (Danvers, MA, USA), while cyclin A, cyclin D1, and p27 antibodies were purchased from Santa Cruz Biotechnology (Santa Cruz, CA, USA). Additional chemicals were obtained from Sigma Chemical Co. (St. Louis, MO, USA).

### 2.2. Preparation of Protein Hydrolysate from Mealworm

Dried mealworms (13–15 instar, 2–2.5 cm), produced following the guidelines of the Korean National Institute of Agricultural Sciences, were purchased from the Yecheon Insect World Farming Association Corporation (Yecheon-gun, Republic of Korea). The mealworms were reared at 25 ± 2 °C and 65 ± 5% relative humidity, with wheat bran as the primary feed and napa cabbage provided as a moisture source. The protein hydrolysates were prepared through enzymatic hydrolysis as previously described [6,13]. Briefly, dried mealworm powder was hydrolyzed at pH 7 and 55 °C using three different proteases—alcalase, flavourzyme, and neutrase—at an enzyme-to-substrate ratio of 1:100 (*w/v*) for 8 h with shaking at 100 rpm (shaking incubator IST-4075R, Jeio Tech Co., Ltd., Daejeon, Republic of Korea). Hydrolysates were filtered to obtain fractions ≤ 1 kDa using a centrifugal filter system (Pall Corp., Port Washington, NY, USA), followed by lyophilization at −110 °C for 72 h (HyperCOOL HC3110, Gyrozen Co., Ltd., Seoul, Republic of Korea).

Detailed methods for the preparation characteristics and quality analysis of mealworm protein hydrolysates are described in Cho and Lee [6].

### 2.3. Differentiation of 3T3-L1 Preadipocytes

3T3-L1 preadipocytes were seeded into plates and induced to differentiate into mature adipocytes. At two days post-confluency (Day 0), cells were treated with a differentiation cocktail (MDI) containing 0.5 mmol/L IBMX, 5 µg/mL insulin, and 1 µmol/L dexamethasone in DMEM for two days. The medium was subsequently replaced every two days with insulin (5 µg/mL). Adipocyte differentiation was confirmed by lipid droplet accumulation, and cells were taken for analysis at 6 days post-induction.

### 2.4. Cell Viability Assay

Cells (1 × 10^5^ cells/well) were seeded in 48-well plates and cultured for six days at 37 °C. Following this, the medium was replaced with 200 µL of differentiation medium containing various concentrations of hydrolysates for the specified time. After treatment, 20 µL of MTT (3-(4,5-dimethylthiazol-2-yl)-2,5-diphenyltetrazolium bromide) solution (2.5 mg/mL) (Amresco, OH, USA) was added to each well, and cells were incubated for an additional 4 h. The resulting formazan crystals were dissolved in 200 µL DMSO, and absorbance was measured using a microplate spectrophotometer (Epoch, Biotek Instruments, Winooski, VT, USA).

### 2.5. Oil Red O Staining

To assess lipid accumulation, 3T3-L1 cells (1 × 10^6^ cells/well) were plated in 6-well plates, treated with MDI with or without hydrolysates, and cultured under the specified conditions. After treatment, cells were gently washed with phosphate-buffered saline (PBS), fixed with 10% formalin for 30 min, and washed three times with water. Cells were stained with an Oil Red O solution (6 parts 0.6% Oil Red O dye in isopropanol to 4 parts water) for 20 min, then washed three times with water. For quantification, the dye was dissolved in isopropanol, and absorbance at 520 nm was measured using a microplate spectrophotometer.

### 2.6. Bromodeoxyuridine (BrdU) Assay

A BrdU assay (Biovision, Milpitas, CA, USA) was used to quantify DNA synthesis in 3T3-L1 cells according to the manufacturer’s protocol. Cells (1 × 10^5^ cells/well) were plated in 48-well plates, and BrdU reagent (1:1000) was added post-differentiation induction for 18 h. Following DNA denaturation, BrdU incorporation was quantified immunochemically, with absorbance measured at 450 nm using a microplate spectrophotometer.

### 2.7. Western Blot Analysis

Cells were harvested using a cell scraper, and cellular lysates were prepared using RIPA buffer (Thermo, Waltham, MA, USA) containing protease and phosphatase inhibitors (Gendopot, Baker, TX, USA). After incubation at 4 °C for 30 min, lysates were centrifuged at 13,000 rpm for 10 min (Labogene 1248R, Bio-Medical Science Co., Ltd., Seoul, Republic of Korea), and supernatants were collected. Protein concentration was determined using a BCA assay kit (Thermo). Equal amounts of protein (25 µg) were resolved via 10% SDS-PAGE, transferred to PVDF membranes, and probed with specific antibodies. Detection was performed using enhanced chemiluminescence, and GAPDH was used as a loading control.

### 2.8. Quantitative Real-Time PCR (qPCR) Analysis

Total RNA was extracted using Trizol reagent (Thermo) in accordance with the manufacturer’s protocol. cDNA was synthesized from RNA (1 µg) using reverse transcriptase with oligo-dT primers and random hexamers. qPCR was conducted on a Thermal Cycler Dice (TP815, Takara, Otsu, Shiga, Japan). The threshold cycle (Ct) values were normalized using *Tbp*, with primer sequences detailed in Table 1.

### 2.9. Statistical Analysis

Data are presented as mean ± SEM (n > 3) per group. Statistical significance was analyzed using Student’s *t*-test or one-way ANOVA with Duncan’s post hoc test (SPSS, version 23.0, Chicago, IL, USA), and *p*-values < 0.05 were considered statistically significant.

## 3. Results and Discussion

### 3.1. Fh-T Promotes Adipogenesis of MDI-Treated 3T3-L1 Cells

**Figure 1:**
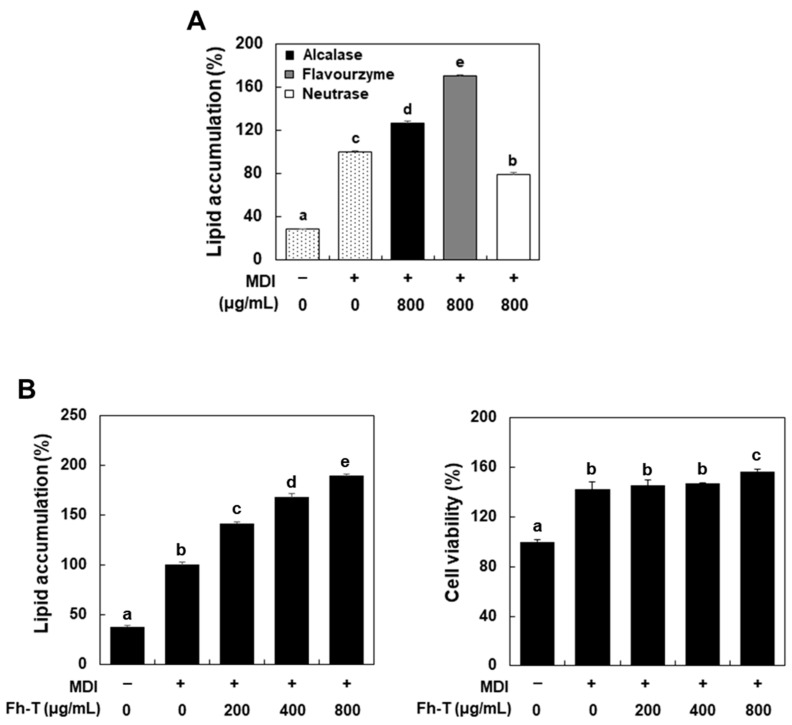
Effect of mealworm protein hydrolysates on adipogenic differentiation of MDI-treated 3T3-L1 cells. (**A**,**B**) 3T3-L1 preadipocytes were cultured as described in the Materials and Methods. Differentiating cells were treated with each protein hydrolysate for 6 days. The results are presented as means ± SEM (n ≥ 3). Different letters above the bars indicate significant differences among groups, according to one-way ANOVA with Duncan’s multiple range test (*p* < 0.05).

In vitro adipocyte hyperplasia can be modeled by adipogenesis in 3T3-L1 preadipocytes, a well-established system for examining preadipocyte differentiation and subcutaneous fat metabolism [14,15,16]. Differentiated 3T3-L1 adipocytes exhibit biochemical and morphological characteristics akin to those of normal adipocytes and show similar responsiveness to metabolic hormones. To assess the adipogenic differentiation effects of protein hydrolysates (<1 kDa) generated using three different enzymes (alcalase, flavourzyme, and neutrase) on 3T3-L1 cells, we treated preadipocytes with 800 μg/mL of these hydrolysates in the presence of MDI for six days. Lipid accumulation was measured through Oil Red O staining, revealing that the treatment with alcalase hydrolysate (Ah-T) and flavourzyme hydrolysate (Fh-T) significantly promoted adipocyte differentiation compared to the MDI-only treatment (Figure 1A). In contrast, the treatment with neutrase hydrolysate (Nh-T) resulted in significantly lower lipid accumulation than the MDI-only treatment, indicating that Nh-T inhibited adipocyte differentiation. Among these, the adipogenesis-promoting effect of Fh-T was markedly higher than that of Ah-T, confirming that Fh-T exhibited the strongest differentiation-promoting effect. Consequently, Fh-T was selected for further analysis of its adipogenic effects and underlying mechanisms.

For Fh-T, slight cytotoxicity was observed in 3T3-L1 cells at concentrations of 1 mg/mL or higher. Therefore, Fh-T was applied at concentrations ranging from 200 to 800 μg/mL, a range that did not exhibit cytotoxicity, to evaluate its adipogenic differentiation-promoting activity (Figure 1B). The results showed significantly higher differentiation activity compared to the MDI-only treatment group at all tested concentrations, with activity increasing in a dose-dependent manner. Additionally, Fh-T demonstrated no cytotoxicity at 200–800 μg/mL and exhibited significantly higher cell viability (>140%) compared to the untreated group in all treatment groups, including the MDI-only group.

MDI treatment enables growth-arrested 3T3-L1 cells to re-enter the cell cycle and undergo MCE in the early stages of adipogenesis, with subsequent upregulation of adipogenic transcription factors and their target genes associated with adipocyte phenotype development [14]. Increased expression of adipogenic transcription factors, such as C/EBPα and PPARγ, is critical for adipogenesis and adipocyte maturation, as these factors stimulate the expression of genes like *Adipoq (adiponectin)*, *Fas*, *Perilipin*, *Acaca*, *Leptin*, and *Scd-1*, all of which play roles in lipid synthesis, transport, and storage [14,17].

**Figure 2:**
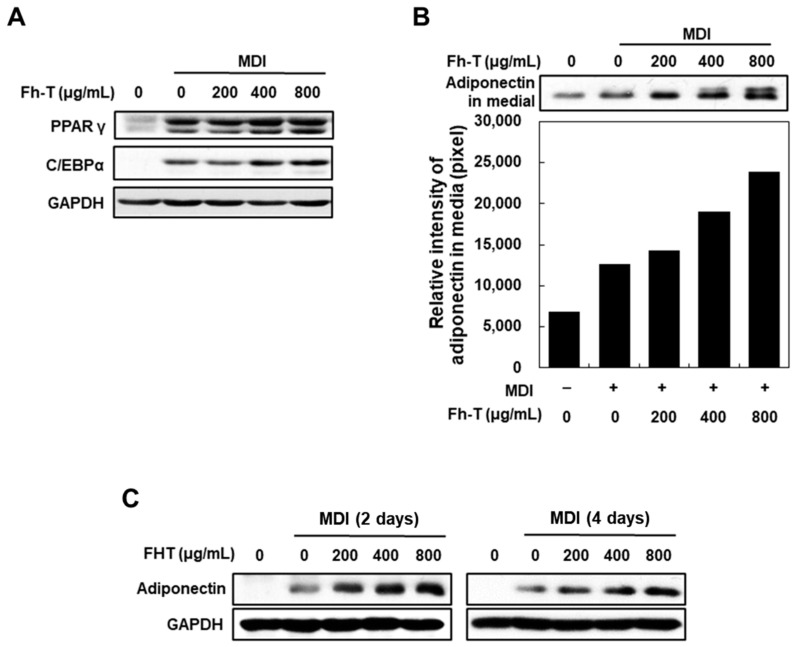
Effect of Fh-T on protein expression of adipogenic markers in MDI-treated 3T3-L1 cells. (**A**,**B**) Cells were treated with different concentrations of Fh-T for 6 days during adipocyte differentiation, and whole-cell lysates (**A**) or medium (**B**) were analyzed by Western blot. GAPDH was used as a loading control. The intensity of the adiponectin bands was measured by ImageJ (version 1.54k). (**C**) Cells were treated with different concentrations of Fh-T for 2 and 4 days during adipocyte differentiation, and cellular adiponectin expression levels were determined by Western blot analysis. GAPDH was used as a loading control.

Following the confirmation that Fh-T promotes adipogenesis in MDI-treated cells, its effects on the expression of key adipocyte markers at both the protein and mRNA levels were further examined. qPCR and Western blot analyses revealed that treatment with Fh-T at concentrations of 400 and 800 μg/mL significantly upregulated the expression of PPARγ and C/EBPα during adipogenesis (Figure 2A). Notably, Fh-T also stimulated adiponectin secretion in 3T3-L1 adipocytes as early as day 2, with a marked increase in cellular adiponectin expression observed by day 4, compared to the MDI-only group (Figure 2B,C). These results suggest that Fh-T induces a robust adipogenic response, potentially stabilizing the adipocyte phenotype.

**Figure 3:**
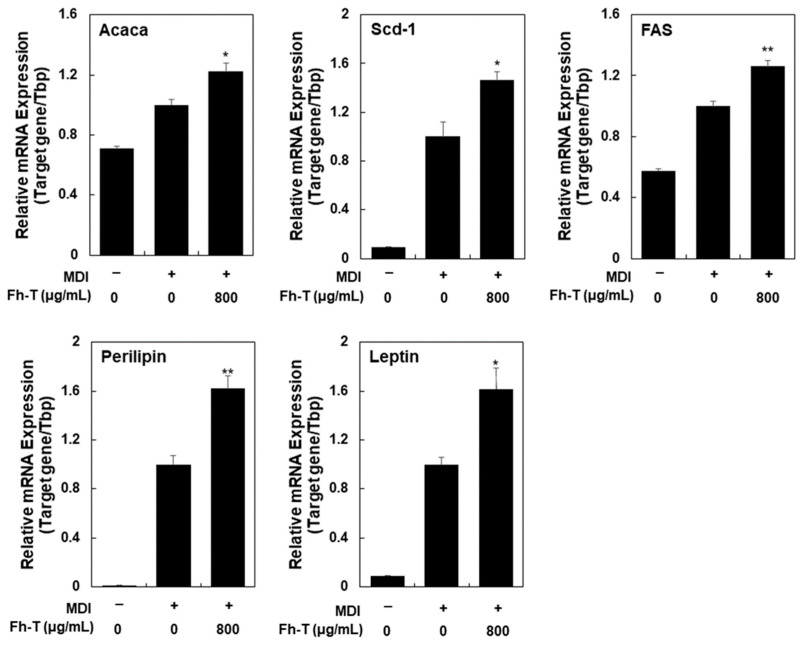
Effect of Fh-T on mRNA expression of adipocyte markers in MDI-treated 3T3-L1 cells. Cells were treated with different concentrations of Fh-T in the presence of MDI for 6 days, and mRNA levels were determined by real-time qPCR, as described in Materials and Methods. Tbp was used as an internal control, and the results are presented as means ± SEM (n ≥ 3). * *p* < 0.05 and ** *p* < 0.01 vs. MDI alone.

To further elucidate its adipogenic effects, we assessed the impact of Fh-T on the mRNA levels of five adipocyte markers—*Fas*, *Perilipin*, *Acaca*, *Leptin*, and *Scd-1*—which are downstream targets of C/EBPα and PPARγ (Figure 3). Treatment with Fh-T (800 μg/mL) significantly enhanced the mRNA expression of these genes in MDI-induced 3T3-L1 cells, highlighting its efficacy in driving adipocyte differentiation and boosting the expression of key adipogenic genes. Collectively, these findings confirm that Fh-T promotes adipocyte differentiation in MDI-treated 3T3-L1 cells within a non-cytotoxic concentration range, demonstrating its potential as a bioactive compound in adipogenesis.

Meanwhile, Seo et al. [11] previously reported that ethanol extracts of mealworm inhibit adipogenesis in 3T3-L1 cells and exhibit anti-obesity effects in a high-fat diet-induced obese mouse model. Similarly, Caldas et al. [18] demonstrated that dietary supplementation with mealworm wholemeal and fermented flour in diet-induced obese mice led to reductions in body weight and adiposity. These findings, which contrast with our results, are likely attributable to differences in the bioactive components of mealworm ethanol extracts, mealworm flour, and mealworm protein hydrolysates. In particular, the adipogenesis-promoting effects observed in our study are presumed to result from the diverse low-molecular-weight peptides present in the protein hydrolysates [6].

Interestingly, recent studies [19,20,21,22,23,24] have consistently shown that protein hydrolysates or low-molecular-weight peptides derived from various plant and animal proteins tend to inhibit adipocyte differentiation. Consequently, findings suggesting that food-derived protein hydrolysates or peptides promote adipogenesis are relatively uncommon.

As demonstrated in Figure 1A, the effect of protein hydrolysates on adipogenesis appears to depend significantly on the type of proteolytic enzyme used during hydrolysate preparation, even when derived from the same protein source. For instance, Nh-T was found to inhibit adipogenesis, suggesting that the bioactivity of low-molecular-weight peptides can vary depending on their composition and structure. These findings highlight the importance of further isolating and characterizing the peptides present in Fh-T. To this end, ongoing research aims to identify and elucidate the specific peptides responsible for these effects.

### 3.2. Fh-T Promotes Adipogenesis of 3T3-L1 Cells Partially Via Regulation of Mitotic Clonal Expansion (MCE)

**Figure 4:**
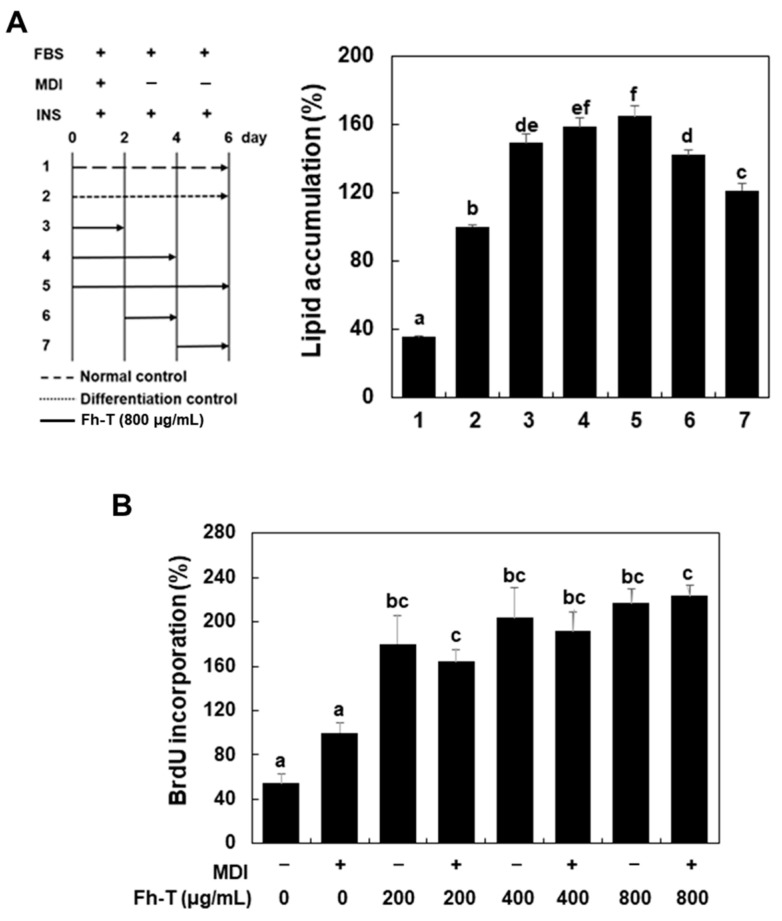
Effects of Fh-T on different stages of adipogenesis and BrdU incorporation in MDI-treated 3T3-L1 cells. (**A**) Schematic model showing Fh-T treatment during adipogenic differentiation of 3T3-L1 cells. The arrows indicate the duration of Fh-T (800 μg/mL) treatment. Cells were stained with Oil Red O at 6 days after the induction of differentiation, and lipid accumulation was measured, as described in the Materials and Methods. (**B**) Growth-arrested cells were treated with Fh-T (800 μg/mL) for 18 h in the presence of MDI, and BrdU incorporation was measured. The results are presented as means ± SEM (n ≥ 3), and different letters above the bars are significantly different at *p* < 0.05 by Duncan’s multiple range test.

During the induction of differentiation, growth-arrested cells re-enter the cell cycle synchronously and undergo MCE in the early stage. This process is followed by the activation of adipogenic transcription factors and their downstream target genes, which drive the development of the adipocyte phenotype [14]. To investigate the mechanism by which Fh-T promotes adipogenic differentiation, we sought to determine the specific stage of differentiation affected by Fh-T treatment. For this purpose, growth-arrested cells were exposed to Fh-T (800 µg/mL) at various time points during the differentiation process, as outlined in Figure 4A (the left panel).

Treatments applied during differentiation days 0–2, 0–4, and 0–6 (treatment plans 3, 4, and 5) resulted in a significant increase in adipogenic differentiation. In contrast, treatments administered at later stages (days 2–4 and 4–6; treatment plans 6 and 7) showed minimal effects (Figure 4A, right panel). These results indicate that Fh-T exerts its adipogenesis-promoting effects primarily during the early phase of differentiation, highlighting its critical role in MCE.

To further explore the mechanism by which Fh-T influences MCE, we assessed its impact on the G1/S transition during the MCE phase in 3T3-L1 cells using a BrdU assay. Growth-arrested cells were exposed to Fh-T in the presence of MDI for 18 h, and BrdU incorporation, a marker of the S phase, was measured. Fh-T treatment significantly increased BrdU incorporation, reflecting enhanced DNA synthesis (Figure 4B). Notably, Fh-T alone was as effective as MDI in promoting MCE (Figure 4B), suggesting that Fh-T facilitates cell cycle progression during the MCE phase in a manner comparable to MDI. However, in the absence of MDI, treatment with Fh-T alone did not induce adipogenesis in 3T3-L1 preadipocytes. This suggests that while Fh-T effectively promotes MCE, its influence diminishes in later stages of adipogenesis. These findings suggest that Fh-T enhances differentiation only when MDI is present, reinforcing its role as a modulator primarily active during the early phase of adipogenesis.

Previous studies [14,17] have shown that successful MCE during adipogenesis requires the expression and activation of various genes, including transcription factors (C/EBPβ and C/EBPδ) and cell cycle regulators (e.g., cyclin A, cyclin D1, and p27). To investigate the impact of Fh-T on these key regulators during the early stage of adipogenesis, protein expression levels were analyzed. Time-course experiments revealed that the temporal expression patterns of these proteins align closely with previously reported findings. Based on these findings, the expression of C/EBPβ and C/EBPδ was analyzed 2 h after MDI treatment, while the expression of cyclin D1, cyclin A, and p27 was examined 18 h post-MDI treatment.

**Figure 5:**
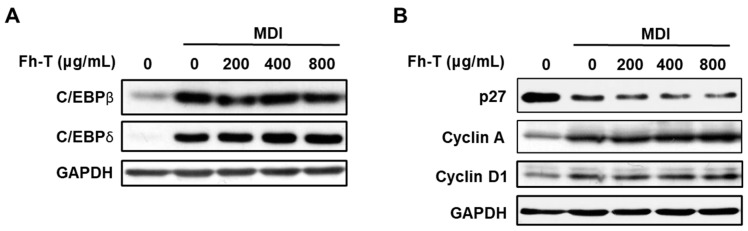
Effect of Fh-T on protein expression of adipogenic transcription factors and cell cycle regulators. Growth-arrested 3T3-L1 cells were treated with Fh-T for 2 h (**A**) and 18 h (**B**) in the presence of MDI. Whole-cell lysates were analyzed by Western blot analysis. GAPDH was used as a loading control.

In 3T3-L1 preadipocytes, MDI treatment rapidly upregulated the expression of C/EBPβ and C/EBPδ proteins, followed by increased cyclin protein levels and decreased p27 expression after 18 h. Co-treatment with Fh-T (400 and 800 µg/mL) further enhanced the expression of C/EBPδ compared to the MDI-only treated cells at the same time points. Additionally, Fh-T significantly reduced the expression of the cell cycle inhibitor p27 (Figure 5). However, Fh-T had a negligible impact on the expression levels of C/EBPβ, cyclin A, and cyclin D1. These findings suggest that Fh-T selectively modulates critical regulatory checkpoints of the cell cycle, supporting its role in enhancing MDI-induced MCE by promoting cell cycle progression through the regulation of C/EBPδ and p27.

The 3T3-L1 adipogenesis model is a valuable tool for discovering functional materials with diverse bioactivities. Specifically, evaluating materials that promote adipogenesis and enhance adiponectin synthesis is used as a screening method for developing agents to improve insulin sensitivity. Moreover, this model is employed to identify skin anti-aging materials by assessing their ability to preserve the subcutaneous fat layer and stimulate the synthesis of type I collagen and hyaluronic acid in dermal fibroblasts [25,26].

The findings of this study contribute to the current understanding of adipogenesis by demonstrating the ability of mealworm-derived protein hydrolysates to promote adipogenic differentiation. Specifically, Fh-T enhances MCE, a critical early stage of adipocyte differentiation, through the regulation of key factors such as C/EBPδ, cyclin A, and p27. These results are consistent with existing knowledge on the importance of MCE in adipogenesis, while also highlighting the unique role of insect-derived bioactive peptides in this process.

The use of mealworm protein hydrolysates represents a novel approach to utilize sustainable and underutilized resources for functional applications. Their ability to stimulate adipogenesis and enhance adiponectin expression suggests potential applications in managing metabolic health, particularly conditions such as insulin resistance. Furthermore, the role of adipokines in skin regeneration, wound healing, and hair growth highlights the potential of Fh-T as a bioactive ingredient for cosmeceuticals and regenerative therapies. These results provide a foundation for further exploration of mealworm-derived ingredients in a variety of fields, helping to bridge the gap between sustainable ingredient sourcing and innovative functional applications.

## 4. Conclusions

This study explored the adipogenic mechanisms of flavourzyme-derived hydrolysates from mealworms (Fh-T) in 3T3-L1 preadipocytes, demonstrating its ability to enhance MDI-induced adipogenic differentiation by significantly promoting MCE. The adipogenic and MCE-promoting effects of Fh-T were linked to the upregulation of C/EBPδ and the downregulation of p27 during the early stages of adipogenesis. These results underscore the potential of Fh-T to stimulate adipogenesis and adiponectin expression, suggesting its broader applicability in adipose-related functions such as glycemic control, skin health, and wound healing. The capacity of Fh-T to modulate key pathways in adipocyte differentiation highlights its promise as a functional ingredient for the development of innovative products in both the food and cosmeceutical industries.

## Figures and Tables

**Table 1:** Sequence of the primers used for qPCR.

Target Gene		Primer Sequence
*Tbp*	Sense	5′-GTGAAGGGTACAAGGGGGTG-3′
	Antisense	5′-ACATCTCAGCAACCC*ACACA*-3′
*Fas*	Sense	5′-AGCACTGCCTTCGGTTCAGTC-3′
	Antisense	5′-AAGAGCTGTGGAGGCCACTTG-3′
*Leptin*	Sense	5′-GAACCTGTCTACTCATGCCA-3′
	Antisense	5′-CTGGTCCTGCAGCCTGTTTG-3′
*Scd-1*	Sense	5′-CAGCCGAGCCTTGTAAGTTC-3′
	Antisense	5′-GCTCTACACCTGCCTCTTCG-3′
*Perilipin*	Sense	5′-GATGAGAGCCATGACGACCAGA-3′
	Antisense	5′-TGTGTACCACACCACCCAGGA-3′
*Acaca*	Sense	5′-GAAGCCACAGTGAAATCTCG-3′
	Antisense	5′-GATGGTTTGGCCTTTCACAT-3′

*Tbp*, TATA-binding protein; *Fas*, fatty acid synthase; *Leptin*, adipocyte-derived hormone; aP2, adipocyte protein 2; *Scd-1*, stearoyl-CoA desaturase-1; *Perilipin*, lipid droplet-associated protein; *Acaca*, acetyl-CoA carboxylase alpha.

## Data Availability

The original contributions presented in the study are included in the article, further inquiries can be directed to the corresponding author.
